# ELK3-ID4 axis governs the metastatic features of triple negative breast cancer

**DOI:** 10.32604/or.2023.042945

**Published:** 2023-11-15

**Authors:** JIN-HO CHOI, JOO DONG PARK, SEUNG HEE CHOI, EUN-SU KO, HYE JUNG JANG, KYUNG-SOON PARK

**Affiliations:** Department of Biomedical Science, CHA University, Seongnam, Korea

**Keywords:** E-cadherin, Extravasation, Colonization

## Abstract

**Purpose:**

Cancer cell metastasis is a multistep process, and the mechanism underlying extravasation remains unclear. ELK3 is a transcription factor that plays a crucial role in regulating various cellular processes, including cancer metastasis. Based on the finding that ELK3 promotes the metastasis of triple-negative breast cancer (TNBC), we investigated whether ELK3 regulates the extravasation of TNBC by forming the ELK3-ID4 axis. ID4 functions as a transcriptional regulator that interacts with other transcription factors, inhibiting their activity and subsequently influencing various biological processes associated with cell differentiation, survival, growth, and metastasis.

**Methods:**

We assessed the correlation between the expression of ELK3 and that of ID4 in TNBCs using bioinformatics analyses, QRT-PCR, western blot analysis, luciferase reporter assays, and chromatin immunoprecipitation. Migration, adhesion, invasion, and lung metastasis assays were employed to determine whether the ELK3-ID4 axis regulates the metastatic features of TNBC.

**Results:**

We found that ELK3 binds directly to a binding motif close to the ID4 promoter to repress promoter activity. The expression of E-cadherin in TNBC was regulated by the ELK3-ID4 axis. *In vitro* and *in vivo* analyses showed that inhibiting ID4 expression in ELK3-knockdown MDA-MB-231 (ELK3KD) cells restored the ability to extravasate and metastasize.

**Conclusion:**

The results indicate that the ELK3 regulates ID4 promoter activity, and that the ELK3-ID4 axis regulates the metastatic characteristics of TNBC cells. Additionally, the data suggest that the ELK3-ID4 axis regulates metastasis of TNBCs by modulating expression of E-cadherin.

## Introduction

Metastasis refers to the dissemination of cancer cells to distant organs, and their subsequent survival in the invaded tissue environment. It constitutes a complex multistep process responsible for 90% of cancer-related deaths [1]. During this process, metastatic cancer cells undergo several biological stages: (1) epithelial-to-mesenchymal transition (EMT), (2) local invasion through the surrounding extracellular matrix and stromal cells, (3) entry into the bloodstream through intravasation, (4) immune evasion, and (5) extravasation to establish new colonies [1–3]. However, only a fraction of the cancer cells that enter the bloodstream from the primary tumor successfully manage to metastasize to other organs. This is attributed to the harsh conditions experienced by cells in the bloodstream, including loss of adhesion and attacks by the immune system. Consequently, the ability of cancer cells to rapidly extravasate into target tissues plays a crucial role in determining their metastatic potential [4,5]. Extravasation of tumor cells involves adhesion to the endothelium, alteration of the endothelial barrier, and successful migration through the endothelial layer into the underlying tissues. Tumor cells can activate mechanisms that directly promote extravasation. For instance, breast cancer cells secrete angiopoietin-like 4, which facilitates extravasation and metastasis [6,7].

Breast cancer represents a significant global health challenge, accounting for a substantial proportion of cancer-related deaths, with a prevalence of 2.26 million cases in 2020 [8]. Among the various subtypes of breast cancer, triple-negative breast cancer (TNBC) stands out. Comprising approximately 20% of new diagnoses, TNBC lacks the expression of estrogen receptor (ER), progesterone receptor (PR), and HER-2. Its aggressive behavior, characterized by high recurrence rates, metastasis, and mortality, underscores the urgency of addressing this challenging subtype. Conventional treatments effective for other subtypes often prove ineffective due to the absence of specific targets, leaving TNBC patients with limited therapeutic options and unfavorable clinical outcomes. Consequently, the investigation of TNBC’s underlying mechanisms and potential interventions remains imperative for enhancing patient outcomes.

ETS Transcription Factor 3 (ELK3) is multifaceted transcription factor implicated in diverse cellular processes, including cell proliferation, apoptosis, differentiation, and migration. It exhibits tissue-specific expression and contributes to the development and functioning of various organs, such as the heart, liver, and kidney [9,10]. Beyond its physiological functions, ELK3 has been associated with various aspects of cancer progression and metastasis, including migration, invasion, EMT, and angiogenesis. In TNBC, the interaction between miR-200a and ELK3 influences the metastatic nature of the disease [11]. ELK3’s involvement in angiogenesis and its regulation of GATA3 expression, a crucial suppressor of metastasis, in breast cancer has also been documented [12,13]. Furthermore, in gastric cancer, ELK3 governs the expression of genes related to extracellular matrix remodeling, thus facilitating the dissemination of cancer cells [14].

E-cadherin operates as a tumor suppressor by upholding an epithelial phenotype in cancer cells [15]. The activation of E-cadherin on the cell surface of circulating tumor cells (CTCs) through a monoclonal antibody impedes the extravasation of CTCs from the vasculature [16]. The expression of E-cadherin is subject to regulation by both transcription factors and epigenetic mechanisms [17]. Alongside established transcriptional repressors like Snail and ZEB1 [18], emerging factors like HIF1α and ELK3 have been identified as transcriptional regulators of *E-cadherin*, particularly in the context of cancer [19,20]. In TNBC, the reduction of E-cadherin is linked to a more aggressive behavior of tumors, including larger size, higher grade, and the spread to lymph nodes [21]. Around 40% of TNBC cases show decreased E-cadherin and a related protein called β-catenin on the cell membrane [22,23]. This suggests that E-cadherin levels could potentially serve as a marker for predicting treatment outcomes in TNBC patients. Additionally, the shift of E-cadherin from the membrane to the cell interior is associated with heightened tumor aggressiveness in TNBC cases [24].

Inhibitor of DNA-binding 4 (ID4), a member of the inhibitor of DNA binding (ID) protein family, features a basic helix-loop-helix structure; however, it lacks a DNA-binding domain. Instead, ID4 forms various complex dimers with transcription factors to intricately regulate the transcription of target genes. ID4 operates as a prodifferentiation factor [25,26] and serves as a tumor suppressor in most cancer cells [27,28]. Although ID4 does not possess a DNA-binding domain, it regulates transcription of target genes such as *ERα* and *Foxa1*, by participating in a large protein complex [29]. For example, in prostate cancer, the epigenetic silencing of the *ID4* promoter is orchestrated by enhancer of zeste homolog 2 [30]. Nevertheless, the precise mechanism governing the regulation of ID4 expression in cancer remains elusive.

In this study, we investigated the role of ID4 in the metastasis of TNBCs within the framework of the ELK3 axis, which acts as a master regulator of EMT in various malignancies, including breast cancer [11,12,31]. We explored the impact of ELK3 as a transcriptional repressor of the *ID4* promoter *in vitro*, and we demonstrated the involvement of the ELK3-ID4 axis in extravasation of TNBCs *in vivo*.

## Materials and Methods

### Cell culture

The TNBC cell lines MDA-MB-231 and Hs 578T were obtained from the American Type Culture Collection (ATCC, Manassas, VA, USA), and MDA-MB-231-GFP-Luc was obtained from Perkin Elmer (Boston, MA, USA). Both MDA-MB-231 and MDA-MB-231-GFP-Luc were cultured in Dulbecco’s modified Eagle’s medium (DMEM; Gibco, Grand Island, NY, USA) supplemented with 10% fetal bovine serum and 1% penicillin-streptomycin (Gibco). Hs 578T was cultured in DMEM (Gibco) supplemented with 10% fetal bovine serum (Gibco), 1% penicillin-streptomycin (Gibco), and 0.01 mg/mL insulin (Sigma, St. Louis, Missouri, USA). All cells were cultured at 37°C/5% CO_2_.

### Knockdown systems and DNA constructs

ShELK3 retroviral plasmids (Dharmacon, Lafayette, CO, USA) were used to generate ELK3-knockdown (ELK3KD) MDA-MB-231 and Hs 578T cells [12]. shID4 lentiviral plasmids (Genecopoeia, Rockville, USA) were used to generate ELK3/ID4 double-knockdown MDA-MB-231 cells. The DNA and siRNA plasmids are described in the supplementary information (Suppl. Table 1).

### Cell migration and invasion assays

Transwell inserts (8.0 µm; Corning, Arizona, USA) were used for migration and invasion assays. For the invasion assay, Matrigel was coated onto the insert (0.5 mg/mL, 50 μl) for 12 h at 37°C. Next, 5 × 10^4^ cells were seeded onto the upper surface of the membrane. The upper chamber was filled with serum-free DMEM and incubated for 24 h at 37°C. For the migration assay, 1 × 10^4^ cells were seeded onto the upper surface of the membrane, and the upper chamber was filled with serum-free DMEM and incubated for 48 h at 37°C. In both assays, the bottom chamber was filled with complete medium, which acts as a chemoattractant. After incubation, cells migrated to the lower surface of the insert were fixed with 4% paraformaldehyde (PFA), stained with crystal violet (Sigma, Darmstadt, Germany), and photographed under an optical microscope (Nikon Corporation, Tokyo, Japan). Cells migrating to the bottom chamber and invading into the inserts were counted.

### Cell adhesion assays

A 96-well plate was coated with Collagen Type 1 (Sigma, Darmstadt, Germany) for 1 h at 37°C. Subsequently, the plate was subjected to a wash using a 0.1% BSA solution, followed by blocking with a 0.5% BSA solution for 1 h at 37°C. After blocking, 2.5 × 10^4^ cells were seeded onto the plate. Cells were incubated for 90 min at 37°C, after which nonattached cells were washed out, and attached cells were fixed with 4% PFA, stained with crystal violet, and photographed under an optical microscope.

### RNA extraction and quantitative RT-PCR

Total RNA was extracted from cells using TRIzol (Invitrogen, Carlsbad, CA, USA), and cDNA was synthesized using the LeGene 1st Strand cDNA Synthesis System (LeGene Biosciences, San Diego, CA, USA). QRT-PCR was performed using TOPreal™ qPCR 2× PreMIX (Enzynomics, Daejeon, Chungnam, Korea) and the CFX Connect Real-Time PCR Detection System (Bio-Rad, Hercules, California, USA). Expression of mRNA was normalized to that of *GAPDH* using the comparative cycle method. The sequences of the primers used are listed in Suppl. Table 2.

### Luciferase assay

ELK3KD MDA-MB-231 cells were transfected with the indicated plasmids using Lipofectamine 2000 (Invitrogen). After 48 h, cells were harvested and lysed in cell lysis buffer (Cell Signaling Technology, Danvers, MA, USA). The Dual-Luciferase Reporter Assay System (Promega, Madison, WI, USA) was used to measure luciferase activity, and firefly luciferase activity values were normalized to the respective Renilla luciferase activity values.

### Western blot analysis

Cells were lysed in cell lysis buffer (Cell Signaling Technology) containing protease and phosphatase inhibitor cocktails (Thermo Fisher Scientific, Rochester, NY, USA). The protein concentration was determined using the BCA assay kit (Thermo Fisher Scientific), and based on this measurement, all samples were subsequently normalized to a concentration of 2 μg/μl. Total 40 μg of proteins were separated by sodium dodecyl sulfate-polyacrylamide gel electrophoresis (SDS-PAGE) and transferred to polyvinylidene difluoride membranes (Bio-Rad). Immunoreactivity was detected using the luminescent image analyzer ImageQuant LAS-4000 (Fujifilm). The antibodies used are listed in Suppl. Table 3.

### Chromatin immunoprecipitation (ChIP) assay

ELK3KD MDA-MB-231 cells were transfected for 24 h with a control plasmid or a Flag-ELK3-expressing plasmid. Transfected cells were fixed in 1% PFA at room temperature for 15 min for crosslinking of genomic DNA and proteins. To neutralize PFA activity, glycine was added to the crosslinked cells and adjusted to a final concentration of 125 mM. The cells were lysed in cell lysis buffer (Cell Signaling) containing a protease-phosphatase inhibitor cocktail (Thermo Fisher Scientific). DNA was fragmented by sonication, and the supernatant was extracted by centrifugation at 15,493 × g for 15 min at 4°C. For immunoprecipitation, the supernatants were incubated at 4°C overnight with protein A/G magnetic beads (Thermo Fisher Scientific) and 2 µg of anti-Flag antibody (M185-3L, MBL, International Corporation, Woburn, MA, USA) or rabbit IgG. DNA-protein complexes were washed in the following solutions in the order listed: 1 × RIPA buffer, 1 × RIPA with 300 mM NaCl, LiCl buffer, and TE buffer. Proteinase K and SDS were added to separate the DNA-protein complexes. Immunoprecipitated DNA was clarified using the phenol/chloroform purification method and then used for ChIP-qPCR. The amount of immunoprecipitated chromatin was calculated as a percentage of the input.

### In vivo animal studies

For short-term lung extravasation model, female NSG mice (NOD-prkdcscid^em1^Il-2rg^em1^) at 7 weeks of age were obtained from JA BIO (Gyeonggi-do, Korea). The mice were used to generate a lung extravasation model of breast cancer cells through the injection of 1 × 10^6^ 231 Con-GFP-Luc cells, ELK3KD-231-GFP-Luc-siNS cells, or ELK3KD-231-GFP-Luc-siID4 cells into the tail vein (n = 3/group). After 3 days, the mice were sacrificed, and their lungs were harvested for analysis.

For long-term lung metastasis model (transient ID4 knockdown), female NSG mice (NOD-prkdcscid^em1^Il-2rg^em1^) at 7 weeks of age were purchased from JA BIO. Metastasis model was generated through the injection of 1 × 10^6^ 231 Con-GFP-Luc cells, ELK3KD-231-siNS-GFP-Luc cells, or ELK3KD-231-siID4-GFP-Luc cells into the tail vein (n = 3/group). The mice were allowed to grow for 28 days, after which their lungs were harvested for further analysis.

For long-term lung metastasis model (stable ID4 knockdown), female NSG mice (NOD-prkdcscid^em1^Il-2rg^em1^) at 7 weeks of age were obtained from JA BIO. For stable ID4 knockdown long-term metastasis model, 1 × 10^6^ 231 Con GFP-Luc cells, ELK3KD-231-shControl (shCon)-GFP-Luc cells, or ELK3KD-231-shID4-GFP-Luc cells were injected into the tail vein of the mice (n = 3/group). The mice were allowed to grow for 28 days, after which their lungs were harvested for further analysis.

For GFP signal analyzing, fluorescence-labeled organism bioimaging instrument (Neo-Science, Suwon, Gyeonggi, South Korea) was used. The lung tissues were embedded in OCT compound (Corning), sectioned on slide-glasses, and fixed with 4% PFA. Harris’ hematoxylin solution (Sigma) and Eosin Y solution (Sigma) were used for H&E staining to visualize the lung tissue.

All animal studies were approved by the Institutional Animal Care and Use Committee of the Laboratory Animal Research Center of CHA University (IACUC210067, IACUC220142). All experiments were performed in accordance with relevant guidelines and regulations.

### Bioinformatics analysis of published databases

Microarray data derived from MDA-MB-231 and ELK3KD cells were obtained from GSM2199548. Gene expression by human breast cancer tissues and normal tissues was validated using GEPIA2 (http://gepia2.cancer-pku.cn/#index), an online database for analyzing the gene expression profiles of tumors and normal samples from The Cancer Genome Atlas (TCGA) and Genotype-Tissue Expression (GTEx) projects [32]. The association between *ID4* expression and overall survival of breast cancer patients was analyzed using the Kaplan–Meier plotter in GEPIA2. Correlation analysis of *ELK3* and *ID4* expression in breast cancer cells was performed using breast cancer cell line microarrays from GEO (accession code GSE41313).

### Statistical analysis

Statistical analysis was performed using GraphPad Prism 7.0 software. Student’s *t*-test was used to calculate *p*-values. Error bars represent the standard deviation (SD). Pearson’s correlation analysis was used to evaluate correlations between expressed genes. *p* < 0.05 was considered statistically significant. All data used in statistical analyses were originated from at least three independent experiments.

## Results

### In silico validation of the association between ELK3 and ID4 expression in breast cancer patients and TNBC cell lines

**Figure 1:**
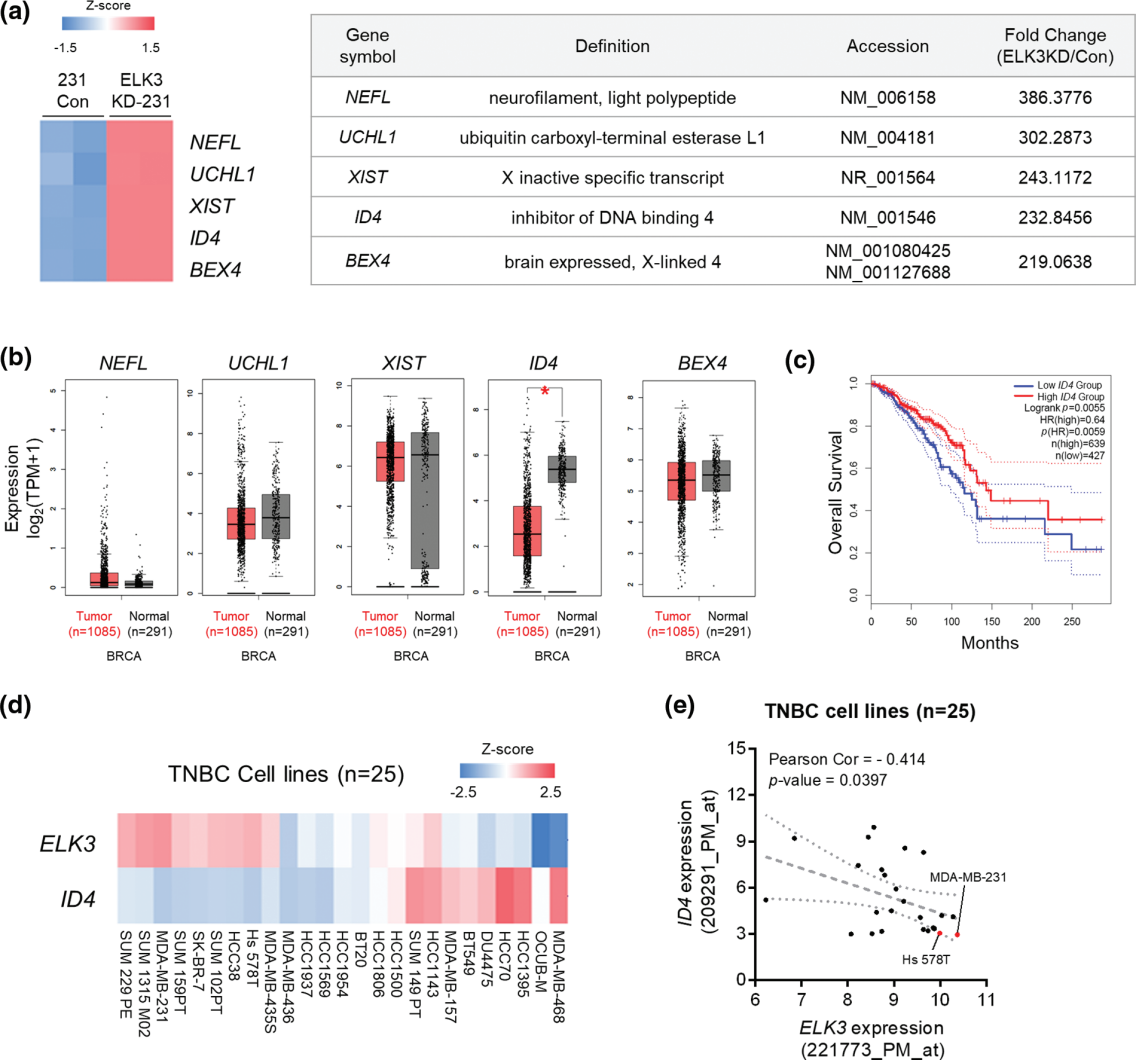
*In silico* validation of the association between *ELK3* and *ID4* expression in breast cancer patients and triple negative breast cancer cell lines. (a) Heat map showing the top five upregulated genes (*NEFL*, *UCHL1*, *XIST*, *ID4*, and *BEX4*) between control and ELK3KD MDA-MB-231 cells (left panel). Expression of the top five upregulated genes is summarized in the table (right panel). Based on a microarray dataset from GSM2199548. (b) Tissue expression levels of *NEFL*, *UCHL1*, *XIST*, *ID4*, and *BEX4* in tumors from breast cancer patients, according to the GEPIA2 database (1085 tumor tissues *vs*. 291 normal tissues). Gene expression analysis based on the box plots was calculated based on the median of log2 (TPM + 1). (c) Kaplan–Meier analysis of overall survival breast cancer patients with high *ID4* or low *ID4* expression (from the GEPIA2 database). Gene expression levels >40% are “high,” and those <40% are “low” (high *ID4*, n = 639; low *ID4*, n = 427). (d) Heat map and (e) correlation graph of *ELK3* and *ID4* mRNA expression levels in 25 TNBC cell lines (obtained from a public microarray dataset: GSE41313). Statistical significance of (e) was calculated based on Pearson’s correlation coefficient. *p < 0.05; n.s., not significant.

Genes regulated by ELK3 were identified by microarray analysis of control and ELK3KD MDA-MB-231 cells. Among these, five candidate genes (*NEFL*, *UCHL1*, *XIST*, *ID4*, and *BEX4)* were upregulated upon ELK3 knockdown (Fig. 1a). We hypothesized that expression of ELK3 target genes may be inversely correlated with ELK3. Therefore, expression of the five selected genes was examined using human breast cancer data from TCGA and GTEx. *ID4* was the only gene whose expression was significantly lower in tumor tissue than in normal tissue (Fig. 1b; 1085 tumor tissues *vs*. 291 normal tissues; analyzed by GEPIA2). Kaplan–Meier plots also indicated that breast cancer patients with low *ID4* expression had significantly poorer overall survival than patients with high *ID4* expression (Fig. 1c; high *ID4*, n = 639; low *ID4*, n = 427; analyzed by GEPIA2). Because ID4 functions as a tumor suppressor in lung adenocarcinoma [33], we examined whether ID4 is a downstream target of ELK3 in TNBC. Bioinformatics analysis of *ELK3* and *ID4* gene signatures in 25 TNBC cell lines showed that TNBC with high expression of *ELK3* have low levels of *ID4* (Figs. 1d and 1e). These data suggest that ELK3 might be a transcriptional repressor of *ID4* in TNBC.

### ELK3 represses the ID4 promoter activity in TNBC

**Figure 2:**
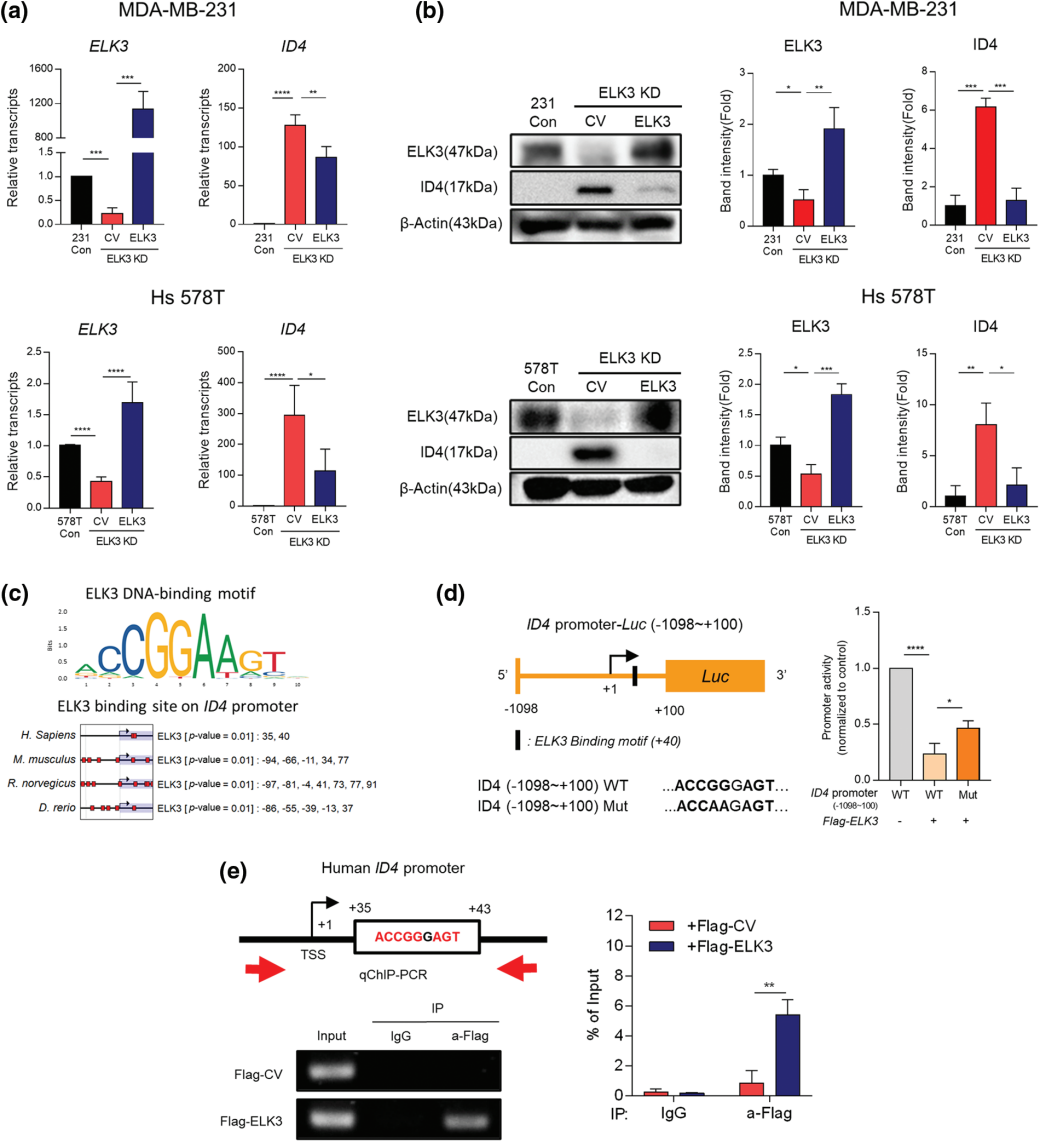
ELK3 represses the transcriptional activity of the *ID4* promoter. Expression of ELK3 and ID4 (a) mRNA and (b) protein in control, ELK3KD, and ELK3KD-231 & 578T cells is restored by introduction of an ELK3-expressing plasmid. (c) ELK3 binding motif on the *ID4* promoter of humans, mice, rats, and zebrafish (cut off, *p*-value < 0.01). Protein-binding motif alignment was performed using EPD (Eukaryotic Promoter Database). (d) (left) Schematic showing construction of the WT *ID4* promoter (−1098~+100 bp) and the Mut ID4 promoter for the luciferase reporter assay. The Mut ID4 promoter has point mutation in the ELK3 binding sequence, as described. (Right) Luciferase assay of the pGL3 reporter plasmid, including the WT *ID4* promoter region (−1098~+100 bp) and the Mut *ID4* promoter. The luciferase reporter plasmid (cloned into the *ID4* promoter) was cotransfected with ELK3-expressing plasmids or control plasmids into ELK3KD MDA-MB-231 cells. (e) ChIP-qPCR analysis of ELK3 binding to the *ID4* promoter region. A Flag-ELK3 expressing plasmid or a Flag-control plasmid was transfected into ELK3KD MDA-MB-231 cells, and Flag-immunoprecipitates were subjected to qPCR using primers specific for the *ID4* promoter region (−28~+107 bp). All data used in statistical analyses were originated from at least three independent experiments. Data represent the mean ± SD. *p < 0.05; **p < 0.01; ***p < 0.001; ****p < 0.0001.

Next, we examined the correlation between ELK3 and ID4 expression in two representative TNBC lines (MDA-MB-231 and Hs 578T) *in vitro*. ID4 was expressed at high levels by ELK3KD, and transfection with an ELK3 expression plasmid reduced expression of both ID4 mRNA and protein to control levels (Figs. 2a and 2b). These data suggest that expression of *ID4* is regulated by ELK3. To confirm that ELK3 binds to the *ID4* promoter to regulate gene transcription, we examined the Eukaryotic Promoter Database for the presence of an ELK3-binding motif on the *ID4* promoter [34]. As expected, an ELK3 binding motif was identified at +34~+41 bp downstream of the transcription initiation site (+1) on the *ID4* gene, and the location of this motif on the *ID4* promoter was conserved in humans, mice, rats, and zebrafish (Fig. 2c). To evaluate whether ELK3 regulates the promoter activity of *ID4* directly, we performed a luciferase reporter assay using a cloned *ID4* promoter. Since the ELK3 binding motif was located about 40 bp downstream of the transcription initiation site (+1), we constructed a WT *ID4* reporter promoter in which the luciferase gene was translationally fused to the region −1098~+100 bp in the *ID4* gene and a Mut *ID4* reporter promoter in which ELK3 binding sequences were mutated. As shown in Fig. 2d, ectopic expression of ELK3 decreased the activity of the WT promoter significantly. Nonetheless, Mut *ID4* promoter activity was decreased to a lesser extent by ectopic ELK3, indicating that ELK3 restricts the activity of the WT *ID4* promoter more stringently than that of the Mut *ID4* promoter. Direct binding of ELK3 to the binding motif at +40 bp in the *ID4* gene was further confirmed by a ChIP assay. Restoring ELK3 expression in ELK3KD-231 using Flag-ELK3 led to a marked increase in ELK3 binding to the corresponding binding motif on the *ID4* gene (Fig. 2e). Taken together, the data suggest that ELK3 binds to the *ID4* gene close to the transcription initiation site and then represses transcription of the gene.

### The ELK3-ID4 axis regulates in vitro migration, adhesion, and invasion of TNBC

**Figure 3:**
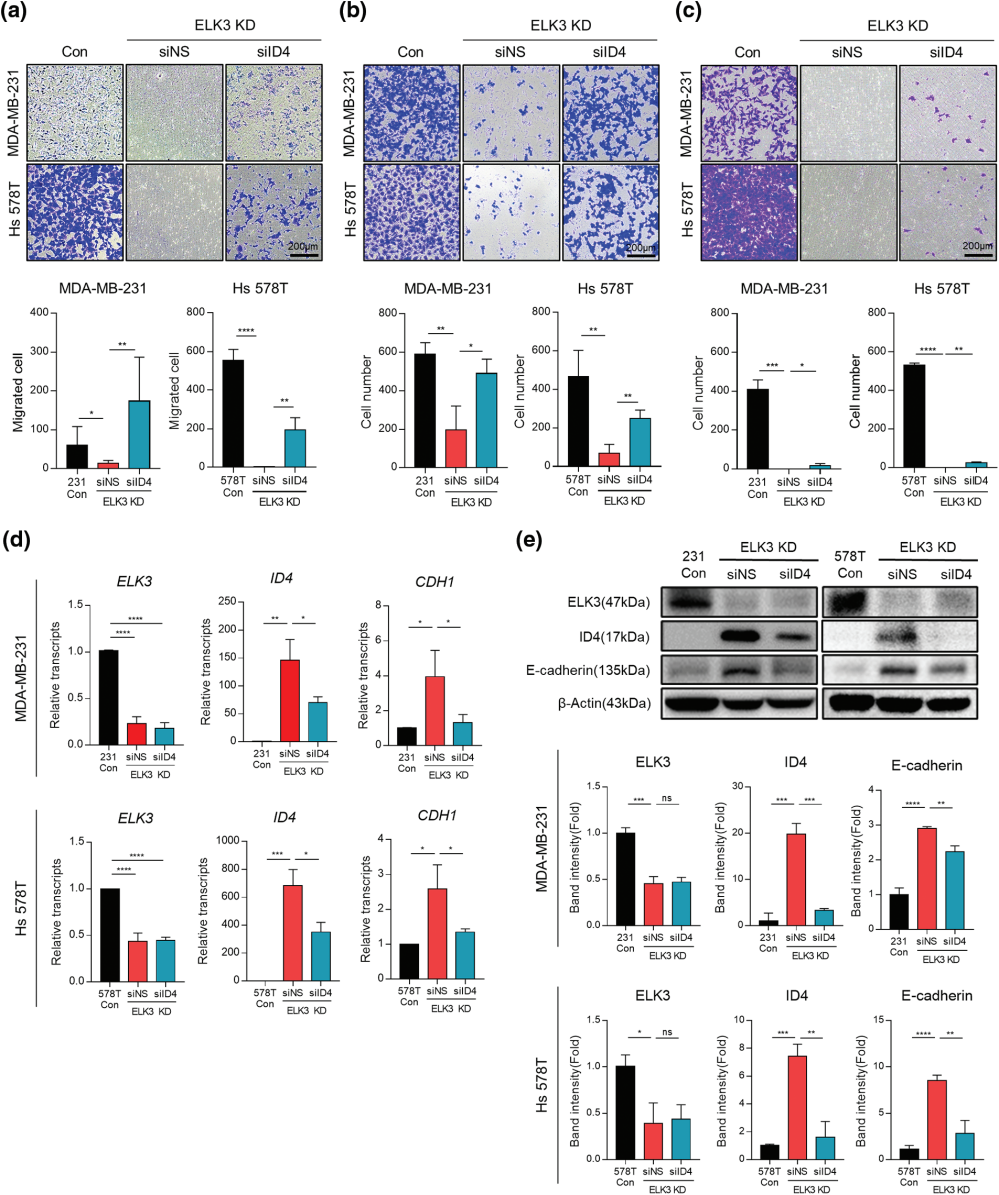
The ELK3-ID4 axis regulates *in vitro* migration, adhesion, and invasion of TNBC. Representative images showing (a) migration, (b) adhesion, and (c) invasion of control and ELK3KD-231 & -578T cells transfected with nonspecific siRNA (siNS) or *ID4*-targeting siRNA (siID4). Quantified graphs are shown under the images. Scale bar, 200 µm. Expression of (d) mRNA and (e) protein products of the indicated genes by control and ELK3KD-231 & -578T cells transfected with nonspecific siRNA (siNS) or *ID4*-targeting siRNA (siID4). All data used in statistical analyses were originated from at least three independent experiments. Data represent the mean ± SD. *p < 0.05; **p < 0.01; ***p < 0.001; ****p < 0.0001.

Inhibition of ELK3 expression by MDA-MB-231 cells reverts their phenotype to epithelial and reduces their metastatic capacity both *in vitro* and *in vivo* [12]. Thus, we investigated the impact of ID4 suppression on migration, adhesion, and invasion of ELK3KD-231 and -578T cells *in vitro* to establish whether the ELK3-ID4 axis is involved in the metastatic features of these cells. When ID4 expression in ELK3KD cells was suppressed by siRNA targeting *ID4* (siID4), migration, adhesion, and invasion showed significant recovery (Figs. 3a–3c). These data indicate that the ELK3-ID4 axis controls the metastatic nature of breast cancer cells *in vitro*. In a previous study [19], we found that the transcriptional repressor complex comprising ELK3 and ZEB1 inhibits transcription of *E-cadherin* in MDA-MB-231 cells, and that ELK3KD-231 cells express more E-cadherin. Given that elevated E-cadherin expression is a hallmark of defective EMT and metastasis [15,35], we investigated whether the ELK3-ID4 axis modulates E-cadherin expression. As shown in Figs. 3d and 3e, transfection of siID4 decreased both the mRNA and protein levels of E-cadherin in ELK3KD cells, further supporting the notion that the ELK3-ID4 axis modulates the metastatic characteristics of TNBCs.

### The ELK3-ID4 axis regulates extravasation and metastatic tumor growth of MDA-MB-231 in vivo

**Figure 4:**
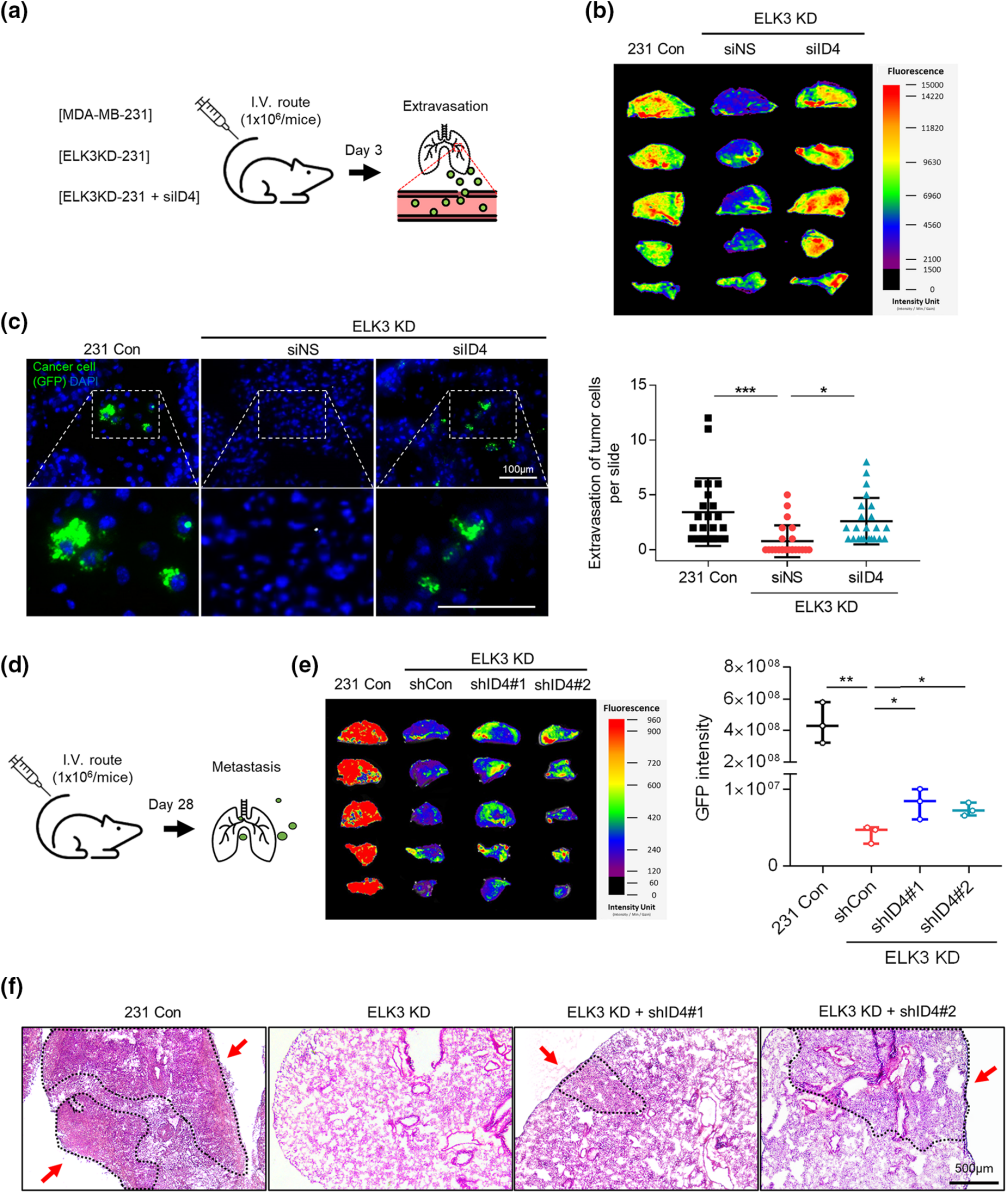
The ELK3-ID4 axis regulates extravasation of breast cancer cells *in vivo*. (a) Schematic of the xenograft and experimental lung extravasation models (n = 3 mice/group). Control MDA-MB-231 GFP-luciferase cells or ELK3KD cells transfected with NS or *ID4* targeting siRNA were injected into NSG mice via the tail vein (1 × 10^6^ cells/mice). (b) Fluorescence images of the lung at 72 h post-tail vein injection. (c) Fluorescence images of GFP, indicating extravasated tumor cells in the lungs of mice. The nuclei were stained with DAPI. Scale bar, 100 μm. The number of GFP-positive cells was counted, and extravasation of tumor cells was determined. (d) Schematic of the xenograft and experimental lung metastasis models (n = 3 mice/group). Control MDA-MB-231 GFP-luciferase cells or ELK3KD cells transduced with NS or *ID4* targeting shRNA were injected into NSG mice via the tail vein (1 × 10^6^ cells/mice). (e) Fluorescence images of the lung at 28 days post-tail vein injection. (f) Sections from mouse lung stained with hematoxylin and eosin. Scale bar, 500 μm. Data represent the mean ± SD. *p < 0.05; **p < 0.01; ***p < 0.001.

Finally, to determine whether the ELK3-ID4 axis in MDA-MB-231 cells has a biological function *in vivo*, we compared the metastatic behavior of ID4-silenced ELK3KD cells with that of control MDA-MB-231 and ELK3KD cells by analyzing extravasation [11]. Control MDA-MB-231, ELK3KD, and ID4-silenced ELK3KD cells engineered to express GFP-luciferase were intravenously injected into highly immunodeficient NSG mice, and the presence of GFP-expressing cells in the lung was examined 3 days later (Fig. 4a). As shown in Fig. 4b, the GFP signal in the lungs of mice injected with ID4-silenced ELK3KD cells was comparable to that in mice injected with control MDA-MB-231 cells, while the GFP signal was barely detected in the lungs of mice injected with ELK3KD. Quantitative analysis of GFP-positive cancer cells in lung tissue frozen sections revealed that ID4-silenced ELK3KD cells reacquired the ability to extravasate (Fig. 4c). These results indicate that the ELK3-ID4 axis regulates extravasation of MDA-MB-231 cells *in vivo*.

**Supplementary Figure 1:**
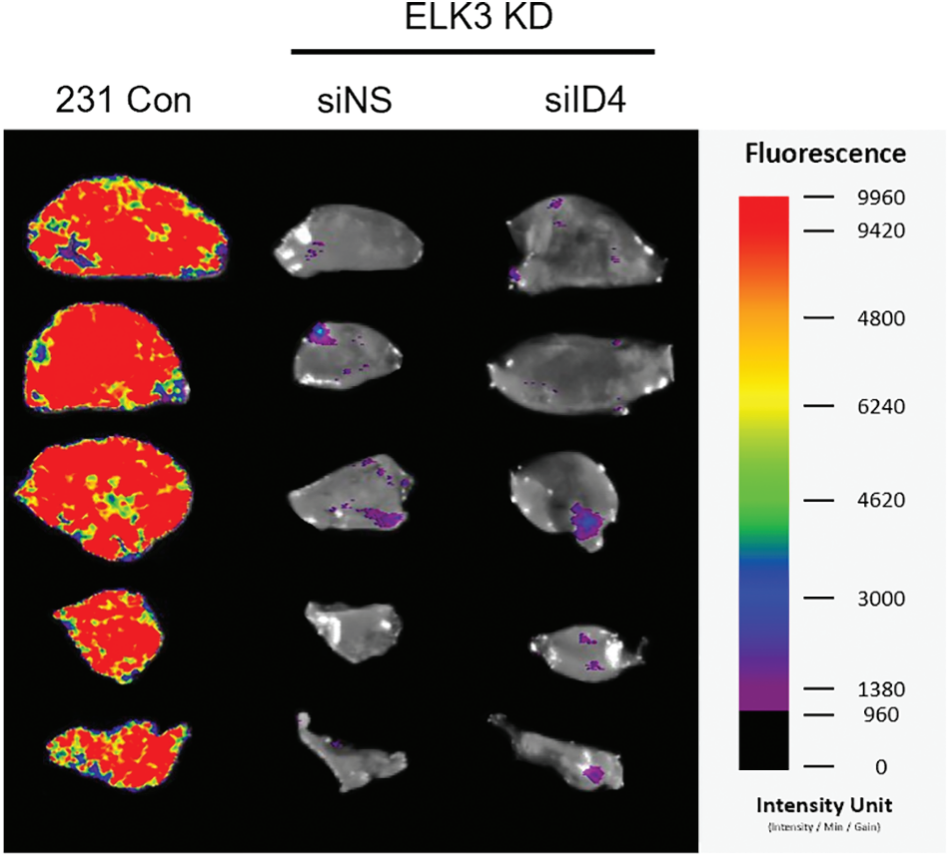
The ELK3-ID4 axis regulates extravasation of breast cancer cells *in vivo*. Fluorescence images of the lung at 28 days post-tail vein injection (siID4).

Subsequently, we conducted long-term lung metastasis experiments to determine whether the extravasated cells form metastatic nodules (Fig. 4d). Although siRNA-mediated suppression of ID4 in ELK3KD cells restored their ability to extravasate, the extravasated cancer cells were unable to form a tumor mass in the lung (Suppl. Fig. 1). To maintain a sustained reduction in ID4 expression even after extravasation, we generated ELK3/ID4KD-231 cells (ELK3KD-231 cells in which ID4 was knocked down permanently). We observed that ELK3/ID4KD-231 cells produced a higher GFP signal than ELK3KD-231 cells (Fig. 4e). Furthermore, tissue analysis through H&E staining revealed that while no metastatic tissue was observed in mice injected with ELK3KD-231 cells, metastatic tissue was observed in mice injected with MDA-MB-231 control cells or ELK3/ID4KD-231 cells (Fig. 4f). These findings suggest that the ELK3-ID4 axis plays an important role in both extravasation and the formation of metastatic cancer tissue.

## Discussion

While extravasation is a crucial step of metastasis, the process remains poorly understood; addressing this issue is essential for the development of effective therapeutic strategies targeting tumor metastasis. In this study, we have demonstrated that the ELK3-ID4 axis is a novel regulatory mechanism controlling the extravasation of TNBC cells. ELK3-mediated repression of ID4 expression is associated with the ability of TNBC cells to extravasate, and alterations in E-cadherin expression might underlie the regulation of extravasation through the ELK3-ID4 axis. Our data suggest that ELK3 indirectly represses E-cadherin expression by acting as a transcriptional repressor of *ID4*.

Unlike ID4, other members of the ID protein family (e.g., ID1 and ID3) are highly expressed in metastatic human breast cancers and exhibit pro-metastatic activity. These molecules initiate invasion during the intravasation step at the primary tumor site and subsequently promote colonization of other organs after extravasation [36–38]. The data clearly indicates that ID4 functions as an inhibitor of extravasation in ELK3KD-231 cells, indicating that different ID proteins play distinct roles in coordinating the multiple factors in cancer cell metastasis.

A recent report demonstrates that ID4 exerts antimetastatic activity in adenocarcinoma by promoting E-cadherin expression [33]. The authors reported that ID4 interacts with Slug, a transcriptional repressor of *E-cadherin*, and sequesters it to increase E-cadherin expression; thus, ID4 induces mesenchymal-epithelial transition in adenocarcinoma. Because Slug also functions as a transcriptional repressor of *E-cadherin* in breast cancer [39], it would be intriguing to ascertain whether the ELK3-ID4 axis is associated with the Slug-E-cadherin axis, in breast cancer or other cancers (including adenocarcinoma).

Considering that various factors, including ANGPTL4, contribute to the extravasation of cancer cells [6], we cannot rule out the possibility that factors beyond E-cadherin are implicated in the ELK3-ID4 axis-mediated extravasation of TNBC. We found that siRNA-mediated suppression of *ID4* in ELK3KD cells restored extravasation competence, and that stable shRNA-mediated suppression of ID4 in ELK3KD cells enabled the formation of a tumor mass in the lung. These data suggest that the ELK3-ID4 axis regulates two major metastatic features-extravasation and colonization-of metastasized cancer cells in distant tissues.

In summary, we identified ID4 as a downstream target of ELK3 in TNBC. We demonstrated that the *ID4* gene contains an ELK3 binding motif near the transcription initiation site (+1), and that ELK3 binds to this motif directly to repress the promoter activity of the *ID4* gene. Furthermore, we also provide evidence that the ELK3-ID4 axis regulates the metastatic nature and extravasation of TNBC both *in vitro* and *in vivo*, which could be mediated through modulation of E-cadherin expression. Our understanding of the metastatic process of cancer cells is limited; the data presented herein will contribute to extending our understanding of this intricate process.

## Supplementary Materials

**Supplementary Table 1:** Plasmids, siRNAs and shRNAs

No.	Name	Information
1	pLenti-cMyc-DDK	Origene, PS1000064
2	pLenti-ELK3-cMyc-DDK	NM_005230.3
3	pRL-TK	Promega, E2231
4	pGL3-basic	Promega, E1751
5	pGL3-hID4 promoter (−1098~+100 bp)	–
6	pGL3-mut hID4 promoter	–
7	Nonspecific siRNA control	Bioneer, SN-1003
8	ID4 siRNA	Bioneer, NM_001546.3
9	Control shRNA	GeneCopoeia CSHCTR001-LVRU6H
10	ID4 shRNA#1	GeneCopoeia NM_ HSH100630-LVRU6H-b
11	ID4 shRNA#2	GeneCopoeia NM_ HSH100630-LVRU6H-c

**Supplementary Table 2:** Primers used

Genes	Forward primer (5′ to 3′)	Reverse primer (5′ to 3′)	Application
*ELK3*	ACC CAA AGG CTT GGA AAT CT	TGT ATG CTG GAG AGC AGT GG	QRT-PCR
*ID4*	GTG CGA TAT GAA CGA CTG CT	CAG GAT CTC CAC TTT GCT GA	QRT-PCR
*GAPDH*	GGG TGT GAA CCA TGA GAA	GTC TTC TGG GTG GCA GTG AT	QRT-PCR
*CDH1*	TGC CCA GAA AAT GAA AAA GG	GTG TAT GTG GCA ATG CGT TC	QRT-PCR
*ID4 promoter* (−28~+107 bp)	ATA AAT ACA GCT GCG CGG CG	TCC CTT CGG AGC TCC GAC TA	ChIP-qPCR

**Supplementary Table 3:** Antibodies used

Antibody	Company	Catalog	Application
ELK3	Novus Biologicals, Centennial, CO, USA	NBP2-01264	Western blot
ID4	Santa Cruz Biotechnology, Dallas, TX, USA	sc-365656	Western blot
β-actin	Santa Cruz Biotechnology, Dallas, TX, USA	sc-47778	Western blot
E-cadherin	Santa Cruz Biotechnology, Dallas, TX, USA	sc-7870	Western blot
Flag	MBL, International Corporation, Woburn, MA, USA	M185-3L	ChIP assay

## Data Availability

Microarray data derived from MDA-MB-231 and ELK3KD cells were obtained from GSM2199548. Gene expression by human breast cancer tissues and normal tissues was validated using GEPIA2 (http://gepia2.cancer-pku.cn/#index), an online database for analyzing the gene expression profiles of tumors and normal samples from The Cancer Genome Atlas (TCGA) and Genotype-Tissue Expression (GTEx) projects [23]. The association between ID4 expression and overall survival of breast cancer patients was analyzed using the Kaplan–Meier plotter in GEPIA2. Correlation analysis of ELK3 and ID4 expression in breast cancer cells was performed using breast cancer cell line microarrays from GEO (accession code GSE41313).
